# Multiscale flow between the branches and polyps of gorgonians

**DOI:** 10.1242/jeb.244520

**Published:** 2023-03-06

**Authors:** Christina L. Hamlet, W. Christopher Strickland, Nicholas Battista, Laura A. Miller

**Affiliations:** ^1^ Department of Mathematics, Bucknell University, Lewisburg, PA 17837, USA; ^2^ Department of Mathematics and Department of Ecology and Evolutionary Biology, University of Tennessee, Knoxville, TN 37996-1320, USA; ^3^ Department of Mathematics and Statistics, The College of New Jersey, Ewing Township, NJ 08628, USA; ^4^ Department of Mathematics, University of Arizona, 617 N. Santa Rita Ave., Tuscon, AZ 85721-0089, USA

**Keywords:** Gorgonian, Computational fluid dynamics, Feeding flows, Sea fans

## Abstract

Gorgonians, including sea fans, are soft corals well known for their elaborate branching structure and how they sway in the ocean. This branching structure can modify environmental flows to be beneficial for feeding in a particular range of velocities and, presumably, for a particular size of prey. As water moves through the elaborate branches, it is slowed, and recirculation zones can form downstream of the colony. At the smaller scale, individual polyps that emerge from the branches expand their tentacles, further slowing the flow. At the smallest scale, the tentacles are covered in tiny pinnules where exchange occurs. In this paper, we quantified the gap to diameter ratios for various gorgonians at the scale of the branches, the polyp tentacles and the pinnules. We then used computational fluid dynamics to determine the flow patterns at all three levels of branching. We quantified the leakiness between the branches, tentacles and pinnules over the biologically relevant range of Reynolds numbers and gap-to-diameter ratios, and found that the branches and tentacles can act as either leaky rakes or solid plates depending upon these dimensionless parameters. The pinnules, in contrast, mostly impede the flow. Using an agent-based modeling framework, we quantified plankton capture as a function of the gap-to-diameter ratio of the branches and the Reynolds number. We found that the capture rate depends critically on both morphology and Reynolds number. The results of the study have implications for how gorgonians modify ambient flows for efficient feeding and exchange.

## Teaser

**Summary:** Extension and retraction of gorgonian polyps can modify their local environment for more efficient feeding and drag reduction.

## INTRODUCTION

**Fig. 1.**
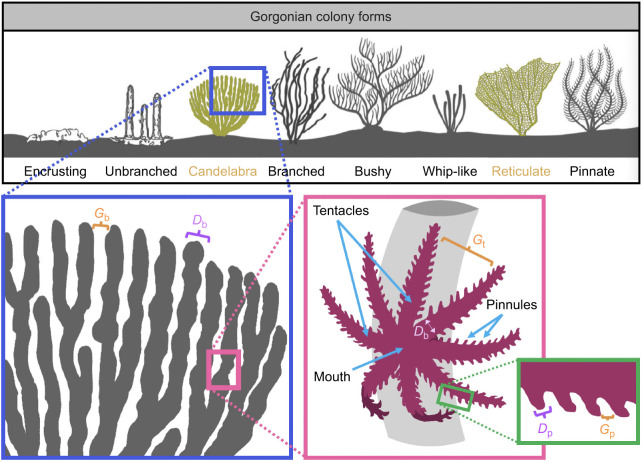
**Schematic of gorgonian colony forms and the morphology of a gorgonian polyp adapted from Kupfner Johnson and Hallock (2020).** In the present study, we focused on gorgonians with branches (including bushy and candelabra) or reticulate morphology. We also focus on the flow at the polyp tentacle and pinnule levels. *G*_b_, *G*_p_, branch or pinnule gap; *D*_b_, *D*_p_, branch or pinnule diameter.

Gorgonians are sessile colonial cnidarians found throughout the oceans of the world that come in a diversity of morphologies and are an integral part of coral reefs (Lesser et al., 2009), especially shallow reef formations (Rossi et al., 2018). The term gorgonian typically refers to octocorals with an internal support axis and branching structure. An internal central skeleton supports the individual polyps (see Fig. 1), and this allows colonies to grow up to 2 m in height, providing habitats and shelter for a variety of organisms (Santavy et al., 2013). Accordingly, gorgonians are thought to provide foundational support for reef communities and are therefore crucial to the health of the reef and the re-establishment of disturbed communities (Lacharite and Metaxas, 2013). Previous work also suggests that these organisms are increasing in abundance and can resist the impacts of climate change (Goulet et al., 2017; Tsounis et al., 2018; Ruzicka et al., 2013; Tsounis and Edmunds, 2017). A variety of colonial forms may be found that depend both on genetics and environment (Prada et al., 2008). Fig. 1 shows the variety of these forms, including encrusting, unbranched, pinnate, whip-like, reticulate and branched (branched, bushy and candelabra) (Bayer et al., 1983). The present study focuses on the flow around branched and reticulate gorgonians. It has been conjectured that these tree-like forms have several advantages, including increased particle capture from the water column and availability of light (Sánchez, 2016).

Gorgonians employ different energy capture strategies depending on the abundance of symbionts and the reliance on foraging (Fabricius and Klumpp, 1995). There are both photosynthetic and non-photosynthetic varieties of gorgonians, with the non-photosynthetic varieties primarily occupying non-photic zones such as caverns or deep-sea environments (Buhl-Mortensen and Mortensen, 2004; Lam and Morton, 2008). Many gorgonian corals have a lower number density of and diversity of prey-capturing nematocysts relative to other cnidarians, and are thought to be mainly dependent on passive suspension capture methods (Lasker, 1981). Gorgonians with planar morphologies often grow such that their fan-like colonial structure is normal to environmental currents to maximize passive suspension feeding (Wainwright and Dillon, 1969; Grigg, 1972). Indeed, laboratory studies using a recirculating flume showed that fan-shaped *Leptogorgia virgulata* colonies capture more *Artemia* per unit time when oriented perpendicular to flow than did colonies oriented parallel to flow (Leversee, 1976).

By measuring feeding rates in different flow conditions, it has been shown that different gorgonians have different optimal flow velocities for prey capture. Dai and Lin (1993) quantified feeding performance as a function of free-stream flow speed for three gorgonians from southern Taiwan, *Subergorgia suberosa*, *Melithaea ochracea* and *Acanthogorgia vegae*. The peak number of *Artemia* captured for all three gorgonians occurred at flow speeds of approximately 8 cm s^−1^. *Subergorgia suberosa*, with the largest polyps, actively fed in a relatively narrow range of flow speeds of 7–9 cm s^−1^. *Melithaea ochracea* and *A. vega* have smaller polyps and actively fed in the range of 4–40 and 2–22 cm s^−1^, respectively. Sponaugle and LaBarbera (1991) measured *Artemia* capture in the Jamaican gorgonian *Pseudopterogorgia acerosa* and found that the capture rate was highest in moderate free-stream flows (10–15 cm s^−1^) and substantially reduced at either lower or higher flow speeds.

Looking at the dynamics of capture at the polyp level, Patterson (1991) observed that prey capture in *Alcyonium siderium* occurs via direct interception. For low flow speeds (<2.7 cm s^−1^), upstream tentacles are more effective at capturing prey. Downstream tentacles have higher capture success at intermediate flows (between 2.7 and 12.2 cm s^−1^), and for higher ranges of flow (>12.2 cm s^−1^), all tentacles have relatively similar capture success. Furthermore, the per polyp capture efficiency drops significantly at higher Reynolds numbers (e.g. higher flow speeds). Capture rates can also vary within the same colony. Coma et al. (2009) found that polyps situated on the apical and peripheral branches of *Paramuricea clavata* captured twice as many prey as polyps in the center or base of the colony. The flow near the polyp, based on streamline measurements along the length of the polyp, has been shown to be 40−80% slower than the free-stream velocity (Sponaugle, 1991), and the configuration of the polyps can alter the flow near them.

Mathematical modeling or numerically simulating the flow through and around branched gorgonians, polyps, tentacles and pinnules is a particularly challenging multiscale fluid dynamics problem. Flow around the entire colony may have a characteristic length scale on the order of a meter, whereas the flow around a single branch or polyp is on the order of a millimeter. When considering the flow between the pinnules, the length scale is on the order of tens of micrometers. Furthermore, highly resolved spatial grids are necessary to numerically resolve such flow fields given highly nonlinear transitions in the character of the flow (Jones et al., 2016). Many organisms that swim, fly, smell or feed in this regime where inertial and viscous forces are balanced often use such branched or bristled structures to take advantage of this nonlinear transition (Koehl, 1995; Cheer and Koehl, 1987; Loudon et al., 1994). These structures can act as either a solid surface or a leaky rake when the characteristic velocity or the spacing between the bristles is changed (Koehl, 2001, 2004). Animals have creative ways of taking advantage of this transition. In the case of tiny insects, the wings are typically bristled and act as solid plates during much of the stroke, but function as leaky rakes to reduce drag when the wings come in contact (Ellington, 1980; Dudley, 2002; Jones et al., 2016). Furthermore, crabs sniff using a bristled appendage that is expanded during the downstroke to allow new fluid to enter and is then contracted during the upstroke to trap the water for sampling (Waldrop et al., 2015, 2016).

The geometries of sea fans may be tuned to take advantage of this leaky-to-solid transition. Slower flows between the branches (e.g. low values of leakiness) could allow for easier prey capture. In contrast, it may be beneficial to be leakier at high flow speeds, such as during intense storms, because this would reduce the drag acting on the sea fan. It is also possible that the geometry is tuned to the local flow environment to enhance feeding efficiency for a particular organism. For example, Weinbauer and Velimirov (1995) quantified the morphological characteristics of fan-like colonies of the gorgonian *Eunicella cavolini*. They measured the porosity (defined here as the percent of open space in the fan area) of colonies from sheltered and exposed habitats and found that sheltered colonies were much more porous. However, they did not determine the effective leakiness of these structures. One open question is whether optimal feeding velocities map to an effective leakiness that is leaky enough to bring food to the colony, but not so leaky that the food cannot be captured as it is quickly advected away.

If one idealizes the geometry of the sea fan colony to an array of cylinders, then it is possible to use classical work in fluid dynamics to better understand the flow at the levels of the branches, tentacles and bristles. Indeed, analytical solutions are available for flows past 2D cylinders in the Stokes regime (negligible inertia) and inviscid regime (zero viscosity) (Batchelor and Batchelor, 1967). Approximations can also be obtained for small Reynolds numbers using Oseen's equation (Proudman and Pearson, 1957). Subsequent work has been done to determine the flow around rows or periodic arrays of cylinders (Fornberg, 1991; Crowdy, 2016, 2006; Ayaz and Pedley, 1999) and pairs of cylinders at low Reynolds numbers (Cheer and Koehl, 1987). By considering a pair of 2D cylinders in an infinite domain, Cheer and Koehl (1987) showed that as Reynolds number increases, leakiness increases for a given gap-to-diameter ratio. At higher Reynolds numbers, the boundary layers around the cylinders are relatively smaller and the shear gradients are steeper, allowing flow between the cylinders. At lower Reynolds numbers, the boundary layers are larger and the leakiness between cylinders is reduced. Similar studies that consider a 2D periodic or infinite array of cylinders cannot be used to examine leakiness in the same way because the fluid has nowhere to go other than between the cylinders. However, such studies are useful in determining the amount of flow driven through the array for a given pressure gradient driving the flow. In the present study, we consider a 3D infinite array of cylinders of finite height so that the fluid can move around the cylinders and leakiness can be determined.

We used computational fluid dynamics to quantify the flow through idealized branched and reticulated gorgonians. In particular, we varied the Reynolds number (*Re*) and the gap-to-diameter ratio (*G*/*D*) for an infinite array of cylinders in the parameter space relevant to sea fans, including the branches of the sea fan, the tentacles and the pinnules on the tentacles. We measured the gap, diameter and length of gorgonian branches, polyp tentacles and tentacle pinnules to obtain biologically relevant parameters using images available in the literature and online. To validate our simplified models, we also performed numerical simulations of flow through a segment of a 3D model of a reticulated gorgonian. To understand the dynamics of prey capture through these cylindrical arrays, we simulated planktonic agents that are advected with the flow and move with some additional random motion. The fraction of agents captured by the cylindrical array was then compared for a range of *Re* and *G*/*D*.

## MATERIALS AND METHODS

### Relevant dimensionless numbers

For comparison across organisms, we will use dimensionless parameters to describe length scales, time scales and the ratio of inertial to viscous forces. Several definitions of the Reynolds number (*Re*) will be useful for our study. We define *Re*=ρ*LU*/μ where *L* is the characteristic length, such as the sea fan width or branch diameter, *U* is the background flow velocity, and ρ and μ are the fluid's density and dynamic viscosity, respectively. It is also helpful to consider the effective Reynolds number at the level of the flow between gorgonian branches (b), tentacles (t) or the pinnules (p) on the tentacles. We define such Reynolds numbers as *Re_X_*=ρ*D_X_U_X_*/μ, where *X* is the structure of interest, *D_X_* is the diameter of this structure and *U_X_* is the relevant free-stream velocity, or the velocity of the fluid moving around the branches, tentacles or pinnules.

Other dimensionless numbers relevant to the flow between the individual branches, tentacles or pinnules are the leakiness, *Le_X_*, the gap to diameter ratio, *G_X_*/*D_X_*, and the height to diameter ratio, *H_X_*/*D_X_*. Note that we measure the gap as the distance between the structures of interest. The leakiness was originally defined by Cheer and Koehl (1987) and describes the ratio of the volumetric flux through an array of cylinders (or branches, tentacles and pinnules) immersed in a viscous fluid divided by the corresponding volumetric flux in an inviscid flow. More specifically, *Le_X_* is given as *Le_X_*=*Q*/(*UA*), where *U* is the free-stream velocity, *A* is the surface area of the region between the structures that is normal to flow and *Q* is the volumetric flow rate between the structures. For an inertial dominated flow, *Le* would be close to 1. In the case of Stokes flow (*Re*<<1), *Le* would be close to zero. The leakiness depends dramatically on the gap-to-diameter ratio, *G_X_*_/_*D_X_*, and the Reynolds number, *Re_X_*.

Another dimensionless number that is relevant to the uptake and/or release of prey and gases such as CO_2_ and O_2_ is the Péclet number (*Pe*). *Pe* describes the ratio of advective transport to diffusive transport for a given substance in flow. The specific *Pe* for the level being considered (e.g. branch, tentacle, pinnule) may be defined as:
(1)

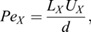
where *L_X_* is the characteristic length, such as the branch diameter, *U_X_* is a characteristic velocity, such as the average flow between branches, and *d* is the diffusion coefficient of the given substance (Vogel, 2013). For *Pe*>>1, advection is the dominant factor in the mass transfer of nutrients and gases. Conversely, for *Pe*<<1, diffusion prevails, and any energy used for active transport of fluid is less effective (Vogel, 2013). Plankton may also be described as having an effective diffusivity if their movement can be approximated as Brownian motion (Kohler et al., 2010). In this case, *Pe* would describe the ratio of advective transport to active motion. For this study, we calculate the Péclet numbers for the diffusivity of plankton (Ozalp et al., 2020), oxygen and carbon dioxide as *d*_p_=2.5×10^–3^ cm^2^ s^−1^, *d*_O_2__=1.97×10^–5^ cm^2^ s^−1^ and *d*_CO_2__=1.60×10^–5^ cm^2^ s^−1^, respectively.

### Parameter measurements

Images of branched and reticulate gorgonians and polyps obtained from the literature were used to determine characteristic lengths and calculate dimensionless numbers. Morphological data were extracted from the images using ImageJ (Schneider et al., 2012). When available, images were calibrated using the known length of a scale bar. In some cases, a provided characteristic length (e.g. the width of the sea fan or diameter of a polyp) was used to calibrate the images. Otherwise, lengths are reported in pixels. For measurements at the branch level, at least five diameter and gap measurements were taken for each section and averaged. Typical measurements for *Euplexaura* and *Swiftia* polyps found in Bayer (1959), Breedy et al. (2019) and Cairns and Wirshing (2015) were used to set the scale for images in ImageJ and to estimate sizes for polyps and bristles.

### Numerical simulations of infinite arrays of cylinders

**Fig. 2.**
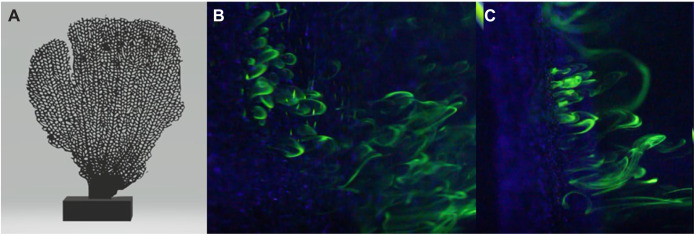
**Qualitative characterization of flow through a physical model of a reticulated gorgonian.** (A) A 3D printed model of a reticulate sea fan. (B,C) Zoomed-in view of the flow between branches using fluorescein dye. The background flow is approximately 10 cm s^−1^. (B) Snapshot taken at a 45 deg angle to the sea fan; (C) snapshot taken nearly orthogonal to the sea fan. Note that the flow between the branches is laminar and relatively steady but becomes turbulent downstream.

This section describes how numerical simulations were performed of one cylinder using periodic boundary conditions to represent an array of cylinders. The simulations were run for a variety of flow and geometric configurations, and leakiness for each simulation was measured. The total parameter space covered two independent variables, *Re* and *G*/*D*. The one dependent variable measured was *Le*. These numerical simulations were performed in COMSOL Multiphysics 4.5. Using the conventions of this software package, a stationary study (e.g. ∂*u*/∂*t*=0) was performed for single-phase laminar flow. This means that the fluid considered was simply a liquid, the flow was not turbulent and the flow field was not allowed to change in time. The range of *Re* considered was from order 1 to 1000, which is characterized by laminar flow. This assumption was justified through dye visualization experiments. Fluorescein was used to visualize the flow through a 3D printed model of a reticulate gorgonian (see Fig. 2) with a background flow speed of approximately 10 cm s^−1^. This resulted in a branch-based *Re*_b_ of approximately 200. We found that the flow between the branches was laminar with no significant unsteady behavior such as alternate vortex shedding or a transition to turbulence (see Fig. 2B,C). Furthermore, we performed unsteady simulations using the full Navier–Stokes equations and found the same values of leakiness within 1%.

The density of the fluid was set to ρ=1.025 g cm^−3^ with a dynamic viscosity of μ=0.0108*P*, within the typical range of seawater. A cylinder was placed in the center of a working section with periodic boundary conditions in the *x*-direction. Note that periodic boundary conditions mean that conditions on the left exactly match the conditions on the right, so that the single window can be viewed as a motif for an infinite pattern extending in the periodic direction. In this way, we can represent an infinite array of cylinders with a computational model of a single cylinder using periodic boundary conditions. The cylinder was positioned 2 cm downstream of the inlet and had a diameter of 0.1 cm and a length of 1 cm. The fluid domain was 10 cm long and 4 cm high, and the width of the domain varied from 0.15 to 0.9 cm to model different gap-to-diameter ratios. The background flow velocity was varied from 0.1 to 100 cm s^−1^ to cover *Re*_b_ ranging from order 1 to 1000. This range of velocities was chosen because the optimal flow speeds for plankton capture are around 2–15 cm s^−1^, depending on the species. Note that the strongest flow considered was 100 cm s^−1^, which is well beyond the feeding range, and would yield an *Re* of approximately 1000. This case was chosen to consider the flow in a strong current when the polyps are likely contracted.

The domain entrance was given an inlet boundary condition of uniform flow, and the back wall was given a zero-pressure outlet condition. As mentioned previously, the side walls (parallel to the central axis of the cylinder) used periodic boundary conditions to represent an infinite array of cylinders. The top and bottom of the domain (normal to the central axis of the cylinder) used symmetry boundary conditions. The fluid domain was discretized using a free tetrahedral mesh of fine resolution calibrated for fluid dynamics. Note that although the branch diameter of a sea fan varies and may be less than 0.1 cm, the range of *Re*_b_ considered covers a small sea fan with 0.025 cm branches in slow, 0.4 cm s^−1^ flow up to a larger sea fan with 0.05 cm diameter branches in strong, 20 cm s^−1^ flows. Furthermore, the diameter to height ratio of the cylinder was set to 10, within the range of the typical diameter to height ratio measured for gorgonians.

For the simulations at the tentacle scale, the tentacle diameter was set to 0.025 cm with a height of 0.25 cm, and the fluid domain was set to be 2.5 cm long and 1.0 cm high. The cylinder was placed in the middle of the working section and 0.5 cm downstream of the inlet. The tentacle diameter and length are within ranges measured for gorgonians. To consider gap-to-diameter ratios ranging from 1.5 to 3, the width of the domain varied from 0.0625 to 0.1 cm. In order to consider a large range of velocities, the inlet velocity was varied from 0.025 cm s^−1^ (representative of a non-leaky array of branches) to 2.5 cm s^−1^ (representative of strong flow near the surface of the branch). This range of velocities was chosen to model free-stream velocities of approximately 0.1 to 10 cm s^−1^, using the fact that the velocity near the polyp is approximately one-fourth the free-stream velocity for these moderate flow speeds (Sponaugle, 1991). The resulting *Re*_t_ varied from approximately 0.25 to 6.

For the simulations at the pinnule level, a cylinder that was 0.025 mm in diameter and 0.25 mm high was placed in the center of the working section, 0.5 mm downstream of the inlet. The diameter and length are within ranges measured for gorgonian pinnules. The fluid domain was set to be 2.5 mm long and 1.0 mm high. The gap-to-diameter ratio was varied from 1.25 to 2.5, resulting in a domain width that varied from 0.05625 to 0.0875 mm. Finally, the background flow speed was varied from 0.5 to 10 mm s^−1^. This range of velocities was chosen to be approximately 40% of the velocity range considered at the tentacle level. This choice resulted in *Re*_p_ ranging from approximately 0.01 to 0.25.

### Flow through a 3D model of a reticulate gorgonian

**Fig. 3.**
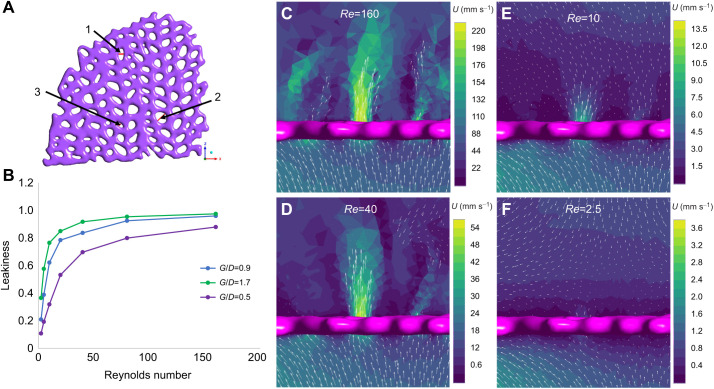
**Numerical simulations of flow through a section of a reticulated gorgonian.** (A) Section of a reticulated gorgonian used for numerical simulation. Leakiness was measured in marked holes (1–3) with *G*/*D*=0.9, 1.7 and 0.5 for holes 1, 2 and 3, respectively. (B) Leakiness as a function of *Re* for the holes 1, 2 and 3. (C–F) Velocity contour in a 2D plane normal to hole 1 with background flows set to 16, 4.0, 1.0 and 0.25 cm s^−1^. The arrows show the direction of flow.

To compare the flow through an actual section of a reticulate sea fan with that through the simplified infinite array of cylinders, we used the same 3D model of a reticulated gorgonian as shown in Fig. 2. Note that a section of the gorgonian was extracted to make the domain's meshing feasible and allow for high spatial resolution for the numerical simulations (see Fig. 3A). The geometry was smoothed for meshing using Autodesk Meshmixer and exported as an STL file. ANSYS 2019 R2 (DeSalvo and Swanson, 1985) was then used to mesh the section of the gorgonian and numerically simulate the steady flow through the branches. Like the COMSOL simulations, a stationary, single-phase flow study was performed using the same fluid density and dynamic viscosity. The computational domain was 100×200×100 mm. The default element size was set to 0.05 mm with adaptive sizing at a resolution of 4.

For this gorgonian model, *G*_b_*/D*_b_ ranges roughly from 0.5 to 2. In order to consider the range of velocities that should result in a transition from leaky to solid, simulations with uniform velocities varied from 2.5 mm s^−1^ to 16 cm s^−1^, yielding branch-based Reynolds numbers, *Re*_b_, ranging from approximately 2.5 to 160. For all simulations, a zero-pressure outlet condition was used. The sides of the domain and the surface of the gorgonian were set to zero-velocity wall conditions.

### The addition of polyps to cylinders

In this section, we take advantage of the idealized geometry of the infinite array of cylinders to consider how the presence of extended polyps affects the flow between branches. As in previous simulations, a 0.1 cm diameter cylinder was used that was 1.0 cm tall. Four idealized polyps were evenly positioned along each side of the cylinder, normal to the direction of flow (see Fig. 6A). The polyps were constructed with eight solid tentacles that were approximately 0.1 cm in length, resting on a stalk that gave the entire polyp a height of approximately 0.09 cm. The pinnules were not included in the model given the challenge of resolving the flow between them and the fact that their leakiness value is small. In all simulations, *G*_b_*/D*_b_ was set to 2.0. *Re*_b_ was varied from 2 to 128. Two values of leakiness were calculated. The leakiness between polyps was calculated by taking the average velocity along a line from the middle of the cylinder to the edge of the domain, and dividing that velocity by the free-stream velocity. The leakiness over the polyps was determined by averaging the velocity along a line taken from the central axis of the second polyp from the top and extending it to the edge of the domain. Both values of leakiness were then compared to the case of a cylinder without polyps.

### Planktos simulations of plankton capture

Our open-source software package, Planktos, was used to simulate the movement and capture of plankton by the filtering array of cylinders (https://github.com/mountaindust/Planktos; Ozalp et al., 2020). For these simulations, the agents move at the local fluid velocity and have an added velocity modeling their active motion. The flow fields generated by COMSOL were imported into the Planktos software package and tiled in the *x*-direction to form a complete flow field 3.6×10×4.0 cm through an array of cylinders. The plankton agents were initialized with random positions in a sheet in the plane [0.4,3.2]×1.0×[0.6,3.4] cm, and they experienced a periodic boundary in the *x*-direction (infinite array of cylinders). The active motion was given as an additional unbiased Brownian motion with a variance of 2.5×10^−3^ cm^2^ s^−1^ (realized by solving a stochastic differential equation using the Euler–Maruyama method), which is representative of plankton such as brine shrimp (Ozalp et al., 2020). All simulations were conducted using 1000 agents with 100 repetitions. Agents were considered captured when they contacted a cylindrical boundary.

## RESULTS

### Flow through a 3D model of a gorgonian

Fig. 3A shows a section of a reticulated gorgonian that was used for numerical simulation. The leakiness was measured in the holes marked as 1, 2 and 3. The leakiness was estimated by averaging the velocity along the red lines and dividing by the free-stream velocity. *G*/*D* was estimated by using the length of the red line as the gap and measuring the diameter of an adjacent section. This results in *G*/*D* of 0.9, 1.7 and 0.5 for holes 1, 2 and 3, respectively. Fig. 3B shows the leakiness as a function of *Re* for each of the holes. As expected, the leakiness is lower for smaller values of *G*/*D* and decreases with decreasing *Re*. For each opening, the leakiness rapidly approaches zero when *Re* is reduced below 40.

Fig. 3C–F shows the velocity contour in a 2D plane normal to hole 1 with background flows set to 16, 4, 1 and 0.25 cm s^−1^. The arrows show the direction of flow and their lengths are proportional to the magnitude. The range of each color map is chosen such that the maximum is set equal to the inlet velocity. The *G*/*D* varies from approximately 0.5 to 2, and the range of branch-based *Re*_b_ is approximately 2.5 to 160. Note that there is significant flow through the hole for the highest *Re* when *U*=16 cm s^−1^. Some additional out of plane jets may also be observed downstream of the gorgonian. As the *Re* is lowered to 40, the strength of the jet is reduced. For *Re*=10, there is minimal flow through the hole and some regions of recirculation can be observed downstream. The jet nearly vanishes for the *Re* case when *U*=2.5 mm s^−1^, and the flow primarily wraps around the sides of the section of the colony.

### Flow through idealized branches, tentacles and pinnules

Table 1 shows the results of the measurements taken at the branch level for 12 individual gorgonians from at least eight genera. The branch-level gap-to-diameter (*G*_b_/*D*_b_) and height-to-diameter (*H*_b_/*D*_b_) ratios are also reported. Diameters are reported in centimeters when length scales are available for the images. Otherwise, the diameters are reported in pixels. The average *G*_b_/*D*_b_ varies from approximately 1.3 to 4. This information was used to inform the parameter space considered in the numerical simulations, where *G*_b_/*D*_b_ was varied more broadly from 0.5 to 8. More variation was observed in the height (or length of a branch between junctions) to diameter ratio, ranging from approximately 4.5 to 30. In all numerical simulations, *H*_b_/*D*_b_ was fixed at 10.

**Table 1.**
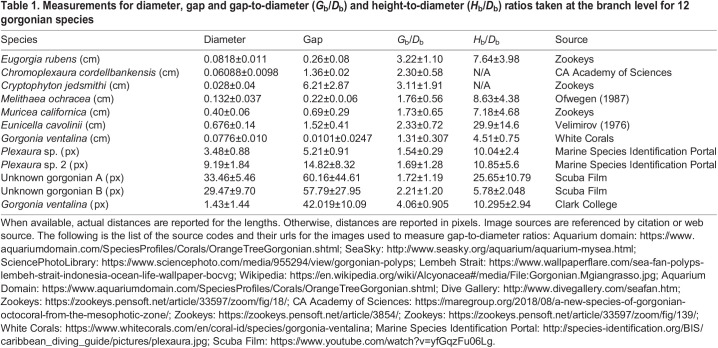
Measurements for diameter, gap and gap-to-diameter (*G*_b_/*D*_b_) and height-to-diameter (*H*_b_/*D*_b_) ratios taken at the branch level for 12 gorgonian species

**Fig. 4.**
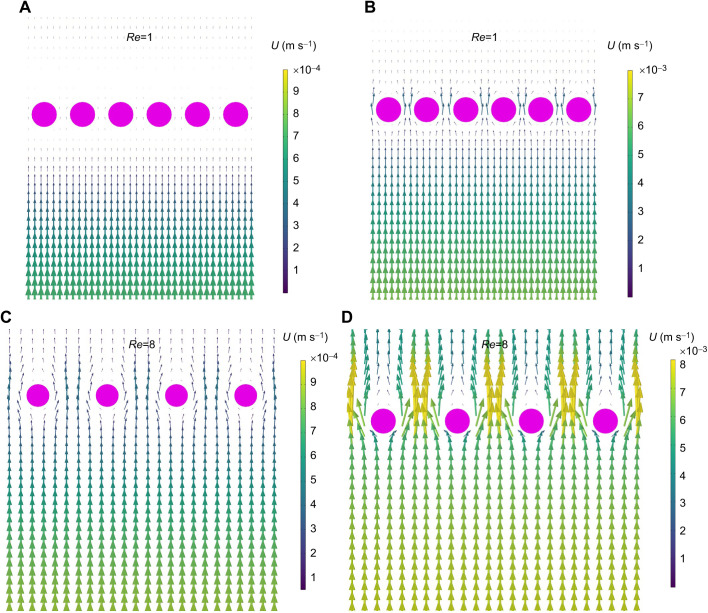
**Velocity vector field displayed in the *x*,*y*-plane cut through the center of the domain.** Flow fields are tiled, taking advantage of periodicity in the *x*-direction. Top row: *G*_b_/*D*_b_=0.5. The inlet velocity is set to (A) 0.1 cm s^−1^ and (B) 0.8 cm s^−1^. The color map is scaled to the inlet velocity in each case. Bottom row: *G*_b_/*D*_b_=2.0. The inlet velocity is set to (C) 0.1 cm s^−1^ and (D) 0.8 cm s^−1^. The color map is scaled to the inlet velocity in each case.

**Fig. 5.**
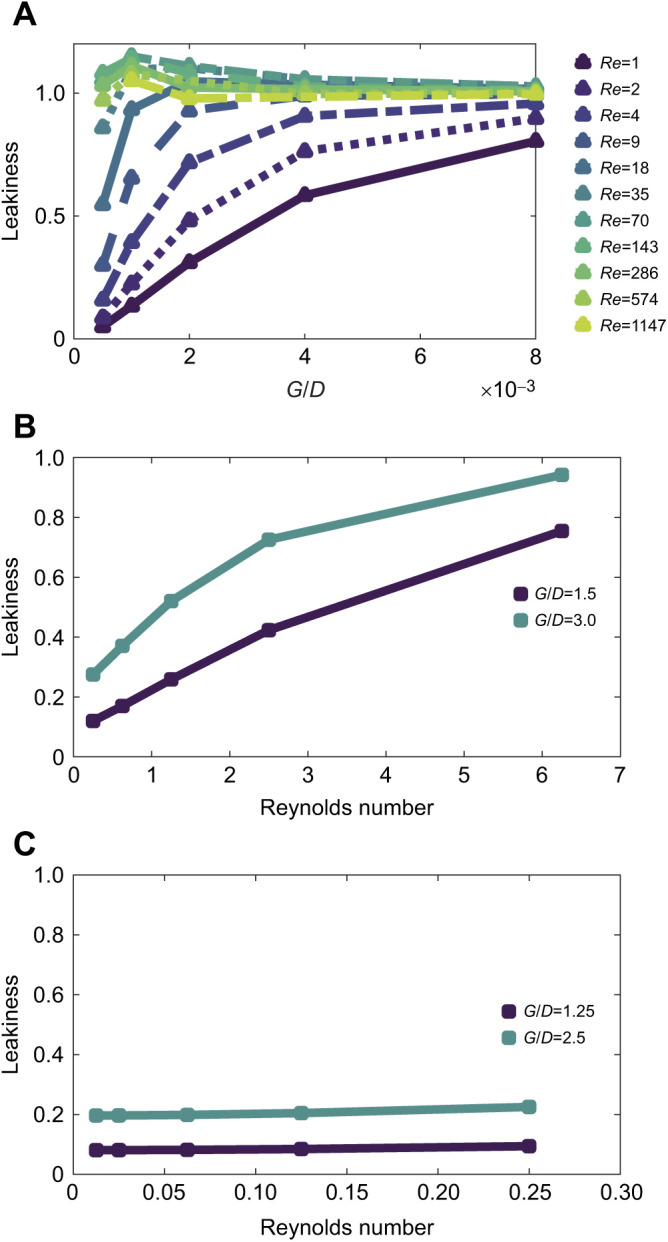
**Leakiness at the branch, tentacle and pinnule levels.** (A) Leakiness as a function of *G*_b_/*D*_b_ for *Re*_b_ ranging from order 1 to 1000. Note that the leakiness in this case is calculated as the average flow between cylinders over the free-stream (e.g. inlet) velocity. (B) Leakiness at the tentacle level, given as a function of *Re*_t_. We assume that the relevant background velocity at the tentacle or polyp level is about one-fourth that of the free-stream velocity. Using a tentacle diameter of 0.25 mm, the tentacle-based *Re*_t_ then ranges from approximately 0.25 to 6.25. *G*_t_/*D*_t_ was set to 1.5 and 3. (C) Leakiness at the pinnule level, given as a function of the pinnule-based *Re*_p_. We assume the relevant background velocity at the tentacle or polyp level is approximately one-half that of the flow around the tentacles, but consider a wide range of speeds (*U*_p_=0.5, 1, 2.5, 5 and 10 mm s^−1^). Using a pinnule diameter of 0.025 mm, the pinnule-based *Re*_p_ then ranges from approximately 0.0125 to 0.25. *G*_p_/*D*_p_ was set to 1.25 and 2.5.

Fig. 5A shows the leakiness as a function of *G*_b_/*D*_b_ for *Re* ranging from order 1 to 1000. Note that the leakiness, in this case, is calculated as the average flow between cylinders over the free-stream (e.g. inlet) velocity. In cases where the flow increases between cylinders due to the reduced cross-sectional area of the fluid domain, the leakiness value is greater than 1. For *Re*_b_ ranging from 1 to 18, there is a sharp transition in the leakiness as *G*_b_/*D*_b_ is decreased from 8 to 1. Note that for all *Re*_b_, there is significant flow between cylinders when *G*_b_/*D*_b_ is 8 or more. For higher *Re*_b_, the leakiness is close to 1 when *G*_b_/*D*_b_ is greater than 1. For *G*_b_/*D*_b_ less than 1, the leakiness sharply decreases, and this transition occurs for smaller values of *G*_b_/*D*_b_ as *Re*_b_ increases. The flow fields for several cases are shown in Fig. 4. The velocity vector fields are displayed in the *x*, *y*-plane cut through the center of the domain along the *z*-axis. The flow fields are tiled in the *x*-direction, taking advantage of the periodicity. The view plane is centered normal to the *x*,*y*-plane, and the cross-sections of the cylinders are shown in magenta. Fig. 4 shows the flow when *G*_b_/*D*_b_=0.5 and the inlet velocity is set to 1 mm s^−1^ (Fig. 4A) and 8 mm s^−1^ (Fig. 4B). The vectors show the direction of flow, and the length of the vectors is proportional to the flow speed. The color map also shows the magnitude of the velocity and is scaled to the inlet velocity. We can observe that there is very little flow between the cylinders when the inlet velocity is *U*=1 mm s^−1^, and this is supported by the low value of leakiness (≈1) in Fig. 4 for *G*_b_/*D*_b_=0.5 and *Re*=1. There is more flow relative to the inlet velocity when *U*=8 mm s^−1^, which is likewise supported by the higher leakiness value (≈0.55) when *G*_b_/*D*_b_=0.5 and *Re*=9. Fig. 4 shows the flow when *G*_b_/*D*_b_=2 and the inlet velocity is set to 1 mm s^−1^ (Fig. 4C) and 8 mm s^−1^ (Fig. 4D). In both cases, there is more flow at larger values of *G*_b_/*D*_b_, with leakiness increasing from approximately 0.3 to over 0.9 as velocity increases from *U*=1 to 8 mm s^−1^.

Table 2 shows the results of the measurements taken at the tentacle level for four genera of gorgonians. The lengths in the table are reported in pixels. In all cases, scale bars were not available. The typical diameter for *Euplexaura* spp. tentacles is approximately 0.47 mm, and the typical diameter for *Swiftia* spp. tentacles is approximately 0.17–0.20 mm. The typical diameter for *Euplexaura* spp. pinnules is approximately 0.089 mm, and the typical diameter for *Swiftia* spp. is approximately 0.030–0.045 mm. This range of length scales was used to determine the appropriate *Re*_t_ range used for the numerical simulations. We see some variation in the gap to diameter ratio at the tentacle level, ranging from approximately *G*_t_/*D*_t_=1.75 to 3. This information was used to inform the parameter space considered in the numerical simulations, where *G*_t_/*D*_t_ was varied from 1.5 to 3. Similar to the tentacle level, we see the variation in pinnule gap-to-diameter ratio, ranging from approximately 1.25 to 2.5. In the numerical simulations, *G*_p_/*D*_p_ was varied from 1.25 to 2.5.

**Table 2.**
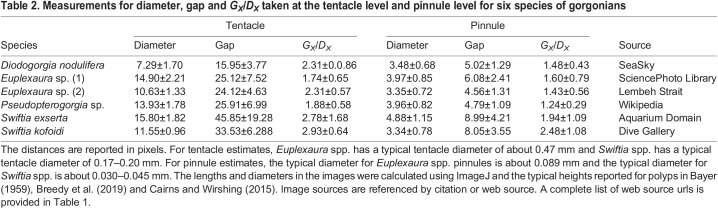
Measurements for diameter, gap and *G_X_*/*D_X_* taken at the tentacle level and pinnule level for six species of gorgonians

Fig. 5B shows the leakiness as a function of the tentacle-based Reynolds number (*Re*_t_) for *G*_t_/*D*_t_=1.5 and 3. Note that this parameter space considers cases of a relatively small tentacle (diameter≈0.25 mm) in a very slow flow (*U*=0.25 mm s^−1^), where the gorgonian at the branch level acts as a solid surface to cases of a larger polyp (tentacle diameter of approximately 0.5 mm) in a stronger flow with a free-stream velocity of approximately 10 cm s^−1^. Over this range of *Re*_t_, the flow through the tentacles transitions from solid to leaky for *G*_t_/*D*_t_=1.5 and 3. This transition suggests that the polyps can strongly alter the flow through the tentacles by adjusting their posture, e.g. spreading out the tentacles or retracting them.

Fig. 5C shows the leakiness as a function of the pinnule-based Reynolds number (*Re*_p_) for *G*_p_/*D*_p_=1.25 and 2.5. Note that this parameter space considers the case when a relatively small pinnule with a diameter of approximately 0.025 mm is in a very slow flow (*U*=0.1 mm s^−1^) owing to the tentacles acting as a solid surface to the case when a larger polyp with a tentacle diameter of approximately 0.1 mm is in a moderately faster local flow of approximately 0.5–1 mm s^−1^. Over this range of *Re*_p_, the leakiness of the bristles is relatively constant, with the larger *G*_p_/*D*_p_ resulting in a relatively leakier bristle array. This suggests the flow through the bristles is only about 10–20% of the magnitude of the flow around the tentacles.

### How the presence of polyps affects flow at the branch level

**Fig. 6.**
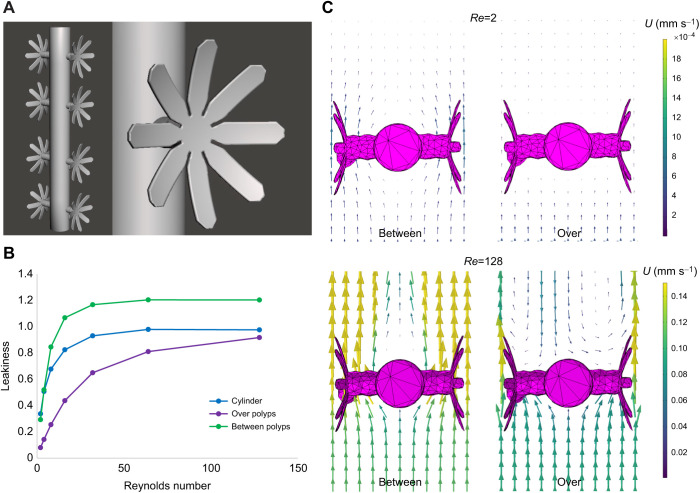
**Simulations of a periodic array of cylinders with polyps.** (A) Idealized models of four octocoral polyps were evenly spaced on each side of the cylinder, normal to flow. (B) Leakiness as a function of Reynolds number for a cylinder without polyps (blue). For a cylinder with polyps, leakiness was measured between polyps (green) and over the polyp (purple). (C) Flow fields taken in planes between the polyps (left) and through the center of polyps (right) with background flow speeds of 0.2 and 12.8 cm s^−1^.

In this section, flow was simulated through a periodic array of cylinders with *G*_b_/*D*_b_=2 and with evenly spaced polyps positioned normal to flow. Fig. 6B shows the leakiness as a function of *Re*_b_ for a plain cylinder and a cylinder with the polyps. Note that the leakiness was measured along a line normal to flow between each polyp (between polyps) and also along a line through the central axis of the polyps (over polyps). The former is representative of how much fluid moves between polyps, whereas the latter is representative of the flow near the oral surface of the polyps. For all *Re* considered, the leakiness over the polyps is much lower than between polyps. It is also much lower than the case of the corresponding plain cylinder. This demonstrates how the presence of the polyps can drastically reduce flow speeds in the range typical of gorgonian feeding. Fig. 6B shows the velocity vector field in a plane positioned horizontally in the center of the cylinder and between polyps (left images) and through the center of the second polyp from the top (right images). For *Re*=2 with a free-stream velocity of ν=0.2 cm s^−1^, there is little flow either between or over the polyps. For the highest *Re* considered, where ν=12.8 cm s^−1^, the flow is still reduced over the polyps relative to the flow between them. Furthermore, there is a recirculation zone downstream of the polyps. Note that the flow speeds are significantly reduced relative to the free-stream velocity, and the flow direction reverses.

### Simulations of plankton capture as a function of *G*_b_/*D*_b_ and *Re*_b_

**Fig. 7.**
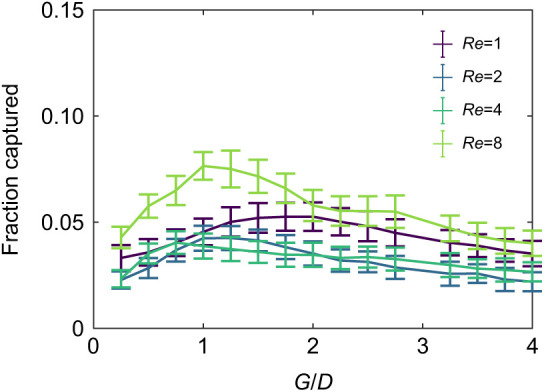
**Average fraction of plankton captured as a function of *G*_b_/*D*_b_ for *Re*_b_=1, 2, 4 and 8.** Error bars show the standard deviations for the average fraction of captured plankton across 100 simulations.

Fig. 7 shows the average fraction of plankton captured as a function of the branched-based *G*_b_/*D*_b_ for the infinite array of cylinders. The error bars show the standard deviation across 100 simulations, and the branch-based *Re*_b_ is varied from 1 to 8. For the highest Reynolds number considered, *Re*_b_=8 and *Pe*_b_=32, the fraction of plankton captured is maximized when *G*_b_/*D*_b_=1. For the lowest Reynolds number considered, *Re*_b_=1 and *Pe*_b_=4, the fraction captured is maximized when *G*_b_/*D*_b_=1.75. For all *Re*_b_ considered, the fraction captured decreases as *G*_b_/*D*_b_ increases to 3 or more. The fraction captured also decreases for *G*_b_/*D*_b_ less than 1. This is in agreement with previous work that shows that there is an optimal, intermediate background flow velocity that maximizes capture (Sponaugle, 1991). For higher flow speeds and structures with high leakiness values, the plankton are swept around the cylinders before they can actively swim through the boundary layer, preventing capture. At intermediate velocities with some leakiness, the plankton are swept between the cylinders, and the flow is slow enough that there is still time for the plankton to move through the boundary layer and allow capture. For the slowest flows with low leakiness, the plankton are swept around the cylinders rather than between them and are not captured. Note that we have only considered lower *Re*_b_ for these simulations. To properly capture the dynamics at higher *Re*_b_, one would need to more carefully model the dynamics of capture. Rather than treating any contact as a capture event, additional physics, such as the time and force required to capture, should be incorporated into the model.

## DISCUSSION

In this paper, we used a simplified computational model of the flow through the branches, tentacles and pinnules of gorgonians to determine how morphology affects fluid flow. At each level, we found that the average ratio of gap distance between structures to the structure's diameter varies from about 1 to 3 (see Tables 1 and 2). We also found that relatively small changes in morphology can lead to large changes in the amount of fluid flowing through the gorgonian colony at the branch and tentacle levels (see Fig. 5). These changes have important implications for how the posture of the polyps and whether the tentacles are extended or contracted can significantly alter the local flow velocities. At the pinnule level, however, the leakiness is relatively constant such that the fluid velocity between pinnules is linearly proportional to the local flow velocity near the tentacles. We then showed more explicitly how the presence of polyps can alter the local flow velocities by simulating cylindrical branches with attached polyps (see Fig. 6). Using an agent-based modeling framework, we found that for a given *Re*_b_, there is an optimal *G*_b_/*D*_b_ that optimizes particle uptake (see Fig. 7). The flow through the branches must be sufficient to bring the plankton near the gorgonian; however, the flow cannot be so strong that the plankton are swept away before they penetrate the boundary layer around the branches and polyps.

Our results also show that flow through a period array of cylinders is a reasonable approximation of the flow through the reticulated gorgonian. It can be seen in Fig. 3B that for hole 2 with *G*/*D*=1.7, the leakiness increases from 0.37 to 0.77 as *Re*_b_ increases from 2.5 to 10. For the periodic cylinder with *G*/*D*=2.0 (see Fig. 6B), the leakiness increases from 0.33 to 0.72 as *Re* increases from 2 to 10. This demonstrates good agreement within the fast transition region. At the higher end of *Re*_b_, hole 1 in the scanned gorgonian has *Le*=0.96 for *Re*=160, and the cylinder has *Le*=0.97 for *Re*=128. Leakiness results for the other holes also show good agreement, as can be found by comparing equivalent *Le* and *G*/*D* in Figs 3B and 5A.

Of particular relevance to our study is the work by Ayaz and Pedley (1999) describing 2D flow through an infinite array of closely spaced cylinders, and the work by Cheer and Koehl (1987) describing 2D flow through a pair of cylinders. Cheer and Koehl (1987) determined the flow between a pair of 2D cylinders for *Re* ranging from 10^–5^ to 0.5 and for 0.3<*G*/*D*<50. Note that this range of *Re* was below the range studied in this paper. They found that the transition from solid to leaky happens at lower *G*/*D* for higher *Re*. For example, they found a sharp transition at *Re*=0.5 as *G*/*D* increases from 2 to 10. Our results are a natural extension of their work as we found a similar transition occurs at lower *G*/*D* for *Re* greater than 1. For example, at *Re*=8, this transition occurs for *G*/*D* between 0.5 and 2 (see Fig. 5A). Ayaz and Pedley (1999) considered steady 2D flow through an infinite array of cylinders for *Re* up to 40 and 2.3<*G*/*D*<10 and quantified the efficiency of particle capture for finite sized particles. They found that *G*/*D* and the radius of the particles are much more important for capture efficiency than *Re*. In this model setup, however, the fluid must go between cylinders and cannot go around them. In the present study, we found that *Re* is an important parameter for finite arrays as the fluid can move around the cylinders rather than between them, lowering capture efficiency (see Fig. 7).

Table 3 shows the *Re* and *Pe* calculated for feeding and exchange currents at the gorgonian colony, branch, tentacle and pinnule levels. *Re* ranges from highly turbulent unsteady flows near *Re*=10^6^ at the level of the entire gorgonian colony to viscous dominated flow around the pinnules where *Re*=10^−2^. Note that the relevant length scale varies over five orders of magnitude, and the relevant velocity scale varies over nearly four orders in magnitude. These variations highlight some of the computational challenges in numerically simulating the combined multiscale problem. In three dimensions, it becomes computationally challenging to handle spatial grids more than 10^3^ mesh widths in each dimension. Given that at least 10 mesh widths would be required to simulate the flow between the pinnules, solving the combined problem would require on the order of 10^6^ mesh widths in each dimension. Different fluid solvers would also be required at each scale. A turbulence model is needed to resolve flow around the entire colony, whereas a Stokes solver would be advantageous at the pinnule scale. At the polyp and branch levels, directly solving the Navier–Stokes equations (as was done in the present study) would be the appropriate choice.

**Table 3.**
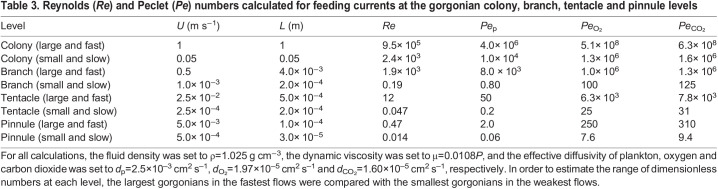
Reynolds (*Re*) and Peclet (*Pe*) numbers calculated for feeding currents at the gorgonian colony, branch, tentacle and pinnule levels

The calculations of *Pe* for plankton capture and O_2_/CO_2_ exchange are also informative. To understand the capture of plankton, *Pe*_p_ uses the effective diffusivity estimated for brine shrimp, where *d*_p_=2.5×10^–3^ cm^2^ s^–1^. For photosynthetic gorgonians, O_2_/CO_2_ exchange is important. To estimate *Pe* for the case of gas exchange, the diffusivity of oxygen was set to *d*_O_2__=1.97×10^–5^ cm^2^ s^–1^, and of carbon dioxide was set to *d*_CO_2__=1.60×10^–5^ cm^2^ s^–1^. At the colony level and for larger branches in faster flows, *Pe*_p_>1 indicates that prey transport is advection-dominated. In the smallest branches and slowest flows, *Pe*_b_<1 indicates that the transport is dominated by diffusion, such that the active transport of the plankton is the most significant factor. At the tentacle level, *Pe*_p_ varies from 0.2 to 50, suggesting that ambient flow and the plankton's active movement can both be significant. The active motion of the prey dominates at the pinnule level. For O_2_/CO_2_ exchange, advection dominates at the levels of the colony, branches and tentacles. Diffusion becomes non-negligible at the pinnule level, where gas exchange is likely to occur.

Given the complexity of this multiscale problem, there are still many aspects of the fluid dynamics to be explored. We have independently investigated the fluid dynamics at the scales of the branches, tentacles and pinnules. We have then quantified how the presence of polyps affects the leakiness between branches for one possible configuration of polyps. It would be worthwhile to further unravel how each scale affects the larger scales. For example, the effective porosity of the colony owing to the morphology of the branches and polyps will alter the flow at the level of the entire colony. In future work, we plan to model the colony as a porous sheet to reveal how leakiness alters the drag acting on the entire colony. The results of that study will be informative in selecting appropriate values of the porosity of the colony for such simulations. One approach to modeling such a porous sheet is described by Kim and Peskin (2006), where a slip between the fluid and the structure, proportional to the porosity of the structure, is incorporated into the numerical method. The porosity, Λ, is a proportionality constant whose physical interpretation is that it is equal to the number of pores in an interval multiplied by the conductance of the material per unit arc length. Note that Λ may be approximated as Λ=(*LeF*)/*U*, where *F* is the steady-state force per unit area acting on a structure with a 90 deg angle of attack to the flow. Additional details of this relation are outlined in Santhanakrishnan et al. (2014).

Similarly, it would be worthwhile to better understand how the flow at the scale of the pinnules and tentacles affects the flow through the branches. Additional studies could be undertaken to reveal how the density, placement and size of the polyps as well as the configuration of the tentacles (e.g. expanded or contracted) affects the flow between the branches. These results could then be used to construct a porosity model that depends upon polyp configuration for whole-colony simulations. Although *Le*_p_ is relatively low, the space between the branches could similarly be modeled as a porous region to understand these effects at the microscale.

We have also assumed that the flow has reached a steady state, and the gorgonian colony is rigid. Unsteady effects could be important as the sea fan sways with the background flow. Furthermore, reaching a steady-state flow between the branches, tentacles and pinnules could take some time. The colony could be operating outside of a steady state for significant portions of each sway. Future work could consider unsteady flows with frequencies relevant to what gorgonians experience in their environments (e.g. the wave frequency). In addition, the flexibility of the gorgonian colony and how it interacts with the unsteady flow could be important. In future work, 3D immersed boundary simulations of this fully coupled fluid–structure interaction problem could reveal the effects of unsteadiness and flexibility. In particular, the angle of flow relative to the colony could change with the addition of flexibility, altering its effective porosity. Admittedly, this is a challenging computational problem that is not feasible as a first approximation to the flow through gorgonian colonies.
